# From Isolation to Integration: How Senior Centers are Poised to Treat the Loneliness Epidemic among Older Adults

**DOI:** 10.1093/geroni/igaf122.3942

**Published:** 2025-12-31

**Authors:** Lauren Roberson, Kimberly Parker, Enid Borden, Sarah Anderson, Susan Zies, Misty Harmon, Sudha Sankar, Abby Pound

**Affiliations:** The Ohio State University, Columbus, Ohio, United States; University of Kentucky, Lexington, Kentucky, United States; National Foundation to End Senior Hunger, Alexandria, Virginia, United States; The Ohio State University, Columbus, Ohio, United States; The Ohio State University, Columbus, Ohio, United States; The Ohio State University, Columbus, Ohio, United States; North Carolina State University, Raleigh, North Carolina, United States; Mid Ohio Food Collective, Columbus, Ohio, United States

## Abstract

As the aging population grows, there is a rise in mental health challenges, including loneliness. Loneliness contributes to decreased quality of life and increased morbidity and mortality. One in three older adults in the U.S. are lonely. As physical places where older adults engage in activities promoting social connection, physical activity, and nutrition, senior centers are an underutilized resource for addressing loneliness. To understand senior center utilization in Ohio, we conducted a cross-sectional survey among older adults aged 60+ who regularly attend senior centers and those who have not within the last three months. Reasons for attending include social support, companionship, recreation, and receiving a nutritious meal. According to both groups, the purpose of a senior center is to provide a place for fellowship, camaraderie, laughter, and life enrichment to keep the golden years active. Among non-users, primary reasons for not attending include a lack of need, interest, and salient programming. Non-users believe senior centers are for “old” people who are clinging to life. This perception could not be further from the truth. ANOVA was used to detect differences in perceptions of senior centers between users and non-users. There were significant differences in perceived importance, with users more likely to agree that senior centers are important. Findings provide insight on the need for tailored outreach to combat the stigma associated with senior centers, their purpose, and the population they serve. This would enable more older adults to benefit from socialization and nutritious meals, improving mental and physical health.

